# Differential Associations of Inflammatory Markers With Insulin Sensitivity and Secretion: The Prospective METSIM Study

**DOI:** 10.1210/jc.2017-01057

**Published:** 2017-07-20

**Authors:** Maria Fizelova, Raimo Jauhiainen, Antti J. Kangas, Pasi Soininen, Mika Ala-Korpela, Johanna Kuusisto, Markku Laakso, Alena Stančáková

**Affiliations:** 1 Institute of Clinical Medicine, Internal Medicine, University of Eastern Finland, Kuopio 70210, Finland; 2 Computational Medicine, Faculty of Medicine, Biocenter Oulu, University of Oulu, Oulu 90014, Finland; 3 Nuclear Magnetic Resonance Metabolomics Laboratory, School of Pharmacy, University of Eastern Finland, Kuopio 70211, Finland; 4 School of Social and Community Medicine, and Medical Research Council Integrative Epidemiology Unit, University of Bristol, Bristol BS8 2BN, United Kingdom; 5 Department of Medicine, Kuopio University Hospital, Kuopio 70210, Finland

## Abstract

**Context:**

Low-grade inflammation is involved in the development of type 2 diabetes and cardiovascular disease (CVD); however, prospective studies evaluating inflammatory markers as predictors of changes in insulin secretion and insulin sensitivity are lacking.

**Objective:**

We investigated the associations of glycoprotein acetyls (GlycA), interleukin-1 receptor antagonist (IL-1RA), and high-sensitivity C-reactive protein (hs-CRP) with insulin secretion, insulin sensitivity, incident type 2 diabetes, hypertension, CVD events, and total mortality in the prospective Metabolic Syndrome in Men (METSIM) study.

**Design:**

A prospective study.

**Participants:**

The cross-sectional METSIM study included 8749 nondiabetic Finnish men aged 45 to 73 years, who had been randomly selected from the population register of Kuopio, Finland. A total of 5401 men participated in the 6.8-year follow-up study.

**Main Outcome Measures:**

Changes in insulin secretion, insulin sensitivity, and cardiometabolic traits during the follow-up period and the incidence of type 2 diabetes, hypertension, CVD events, and total mortality.

**Results:**

During the follow-up period, GlycA was associated with impaired insulin secretion, hyperglycemia, incident type 2 diabetes (hazard ratio, 1.37; 95% confidence interval, 1.29 to 1.46) and CVD (hazard ratio, 1.21; 95% confidence interval, 1.12 to 1.32). IL-1RA and hs-CRP were associated with adverse changes in insulin sensitivity and obesity-related traits and with total mortality (hazard ratio, 1.13; 95% confidence interval, 1.07 to 1.20; and hazard ratio, 1.08; 95% confidence interval, 1.04 to 1.11, respectively).

**Conclusions:**

Inflammatory markers differentially predicted changes in insulin secretion and insulin sensitivity. GlycA predicted impaired insulin secretion, and IL-1RA and hs-CRP predicted changes in insulin sensitivity. Combining the three markers improved the prediction of disease outcomes, suggesting that they capture different aspects of low-grade inflammation.

## Teaser

GlycA, IL-1RA, and hs-CRP were differentially associated with changes in insulin secretion, insulin sensitivity, and incidence of type 2 diabetes and CVD events during the follow-up of the METSIM cohort.

The incidence and prevalence of type 2 diabetes and diabetes-related complications have been consistently increasing worldwide; therefore, identifying those individuals at high risk of diabetes is of great importance. Low-grade systemic inflammation is often associated with insulin resistance and impaired insulin secretion, the two key mechanisms underlying the pathophysiology of type 2 diabetes (1, 2). Elevated levels of acute-phase proteins, such as high-sensitivity C-reactive protein (hs-CRP) and tumor necrosis factor-*α* are often already present in those with prediabetes (3, 4) and are predictive of future type 2 diabetes (5–7) and cardiovascular disease (CVD) events (8, 9). Also, members of the interleukin-1 (IL-1) cytokine family, including IL-1 receptor antagonist (IL-1RA), have been found to play a role in the pathogenesis of type 2 diabetes by triggering obesity-induced inflammation and exacerbating insulin resistance (10). Additionally, IL-1RA levels have been shown to increase gradually with worsening glycemia. In contrast, hs-CRP increased only within the prediabetic range of glucose levels (11). Recently, serum glycoprotein acetylation levels, originating from the *N*-acetyl methyl groups of *N*-acetylglucosamine residues, have been found as a unique biomarker of systemic inflammation, because it captures both circulating levels and complex glycosylation patterns of the most abundant acute-phase proteins (12). The biomarker glycoprotein acetylation correlates strongly with other markers of inflammation (13, 14). In apparently healthy individuals, glycoprotein acetyls (GlycA) can be chronically elevated for periods of up to one decade, suggesting a prolonged low-grade inflammatory state (15). Elevated concentrations of GlycA have been found in patients with type 2 diabetes (16, 17). In large, prospective cohort studies, increased levels of GlycA have been associated with type 2 diabetes (12, 18), coronary heart disease (19, 20), and all-cause mortality, independently of traditional risk factors (21, 22). Additionally, elevated levels of GlycA have been associated with the deterioration of hyperglycemia in a 6.5-year follow-up study of nondiabetic subjects (23) and with insulin resistance in a cross-sectional study of 7098 young Finns (24). However, no large population-based prospective studies have evaluated GlycA as a predictor of changes in insulin secretion, insulin sensitivity, or insulin resistance-related traits. The aim of our 6.8-year follow-up study was to investigate the associations of three acute-phase proteins, GlycA, hs-CRP, and IL-1RA, with the changes in insulin sensitivity and insulin secretion in a large cohort of men without known type 2 diabetes. Additionally, we investigated the associations of these markers with insulin resistance–related traits, incident type 2 diabetes, drug-treated hypertension, CVD events, and total mortality.

## Materials and Methods

### Subjects

The Metabolic Syndrome in Men (METSIM) study included 10,197 Finnish middle-aged men randomly selected from the population register of Kuopio, Eastern Finland (population, 105,000). The baseline study was performed from 2005 to 2010 at the Clinical Research Unit, University of Eastern Finland, Kuopio, Finland. The main study has been previously described in detail (25). The present study included 8749 men without type 2 diabetes at baseline [mean age, 57.2 ± 7.1 years; body mass index (BMI), 26.8 ± 3.8 kg/m^2^]. Their baseline clinical and metabolic characteristics are presented in Table 1 as the means ± standard deviations. Glucose tolerance was evaluated using a 2-hour oral glucose tolerance test (OGTT) with 75 g of glucose after a 12-hour overnight fast, and glucose tolerance status was classified according to the American Diabetes Association criteria (26). Of the 10,197 men, 3034 (34.7%) had normal glucose tolerance, 4344 (49.7%) isolated impaired fasting glucose, 312 (3.6%) had isolated impaired glucose tolerance, and 1059 (12.1%) had both impaired fasting glucose and impaired glucose tolerance. Men with type 1 diabetes (n = 25), previously diagnosed type 2 diabetes (n = 763), type 2 diabetes diagnosed at baseline (n = 649), or missing OGTT data (n = 11) were excluded from the analyses. The ethics committees of the University of Eastern Finland and Kuopio University Hospital approved the present study, which was conducted in accordance with the Declaration of Helsinki. All study participants provided written informed consent. Two datasets of the METSIM study were used in the prospective analyses.

**Table 1.** **Clinical and Metabolic Characteristics of 8749 Men Without Type 2 Diabetes in the Baseline METSIM Study**

Baseline Study Variable	Men, n	Mean ± SD	IQR
Age, y	8749	57.18 ± 7.07	51.00–63.00
BMI, kg/m^2^	8746	26.82 ± 3.81	24.31–28.86
Waist circumference, cm	8745	97.43 ± 10.60	90.00–103.50
Fat mass, %	8727	23.41 ± 6.31	18.90–27.20
Systolic BP, mm Hg	8749	137.16 ± 16.25	126.00–146.67
Diastolic BP, mm Hg	8749	87.21 ± 9.26	80.67–92.67
LDL cholesterol, mmol/L	8746	3.37 ± 0.88	2.76–3.92
HDL cholesterol, mmol/L	8747	1.46 ± 0.39	1.17–1.67
TAG, mmol/L	8749	1.40 ± 0.96	0.88–1.66
ApoA1, g/L	8747	1.41 ± 0.24	1.25–1.55
ApoB, g/L	8747	1.04 ± 0.27	0.85–1.19
Adiponectin, μg/mL	8747	7.94 ± 4.37	5.30–9.50
ALT, U/L	8749	31.31 ± 20.19	20.00–37.00
FPG, mmol/L	8749	5.71 ± 0.48	5.40–6.00
2hPG, mmol/L	8749	6.05 ± 1.69	4.90–7.00
Glucose AUC, mmol/L × min	8719	886.92 ± 136.65	792.00–973.50
HbA1c, %	8727	5.67 ± 0.33	5.50–5.90
InsAUC_0–30_/GluAUC_0–30_	8703	31.10 ± 21.51	17.22–38.43
Matsuda ISI	8697	6.91 ± 4.15	3.79–9.02
Disposition index*^a^*	8697	163.51 ± 71.90	113.37–197.96
GlycA, mmol/L	8716	1.36 ± 0.23	1.21–1.47
hs-CRP, mg/L	8748	2.02 ± 4.04	0.52–2.28
IL-1RA, µg/L	8748	0.21 ± 0.17	0.13–0.25

Abbreviations: ALT, alanine aminotransferase; IQR, interquartile range; SD, standard deviation.

^a^ Disposition index was calculated as Matsuda ISI × InsAUC_0–30_/GluAUC_0–30_.

#### Data for prospective analysis of glycemic traits

A prospective follow-up study was started in 2010. To date, 5401 men without diabetes at baseline or during the follow-up period have been re-examined. The study protocol and measurements were identical to those of the baseline study.

#### Data for prospective analysis of incident type 2 diabetes, incident hypertension, CVD events, and total mortality

Of the 8749 nondiabetic men at baseline, 693 developed incident type 2 diabetes between the baseline and follow-up studies. The diagnosis of new-onset type 2 diabetes was based on the following: (1) fasting plasma glucose (FPG) ≥7.0 mmol/L), 2-hour plasma glucose (2hPG) level in an OGTT of ≥11.1 mmol/L or glycated hemoglobin (HbA1c) of ≥6.5% (diagnosing 327 new cases of diabetes) among nondiabetic men who participated in the follow-up study; (2) antidiabetic medication started between the baseline study and follow-up study (revealing 261 new cases of diabetes; information obtained from the National Drug Reimbursement registry for all 8749 nondiabetic men); or (3) type 2 diabetes diagnosed by a physician according to the medical records and/or an FPG of ≥7.0 mmol/L, 2hPG of ≥11.1 mmol/L, or HbA1c of ≥6.5% in outpatient or primary care laboratory measurements (diagnosing 37 new cases of type 2 diabetes). The follow-up time was calculated as the time in months between the date of the METSIM baseline visit and the date of the METSIM follow-up visit or between the date of the METSIM baseline visit and the start date of antidiabetic medication or the date of the diagnosis of diabetes.

Of the 8749 nondiabetic men, 439 experienced a CVD event during the follow-up study. An incident CVD event was defined as nonfatal myocardial infarction, coronary heart disease, death, or fatal and nonfatal cerebral infarction that had occurred between the baseline and follow-up studies. CVD events were defined according to the internationally accepted criteria (27, 28), identified from hospital discharge registry, and verified from the hospital medical records. Participants with a CVD event before the baseline visit were excluded from the statistical analyses. Of the 8749 nondiabetic men, 225 men started new antihypertensive treatment after the baseline visit, and 392 men died during the follow-up period. These data were obtained from the National Drug Reimbursement registry and the Finnish mortality registry, respectively. Nondiabetic men receiving antihypertensive treatment (n = 1537) at the baseline study were excluded from the statistical analyses evaluating the associations of acute-phase proteins (GlycA, IL-1RA, and hs-CRP) with the development of drug-treated hypertension.

The average follow-up period (6.8 ± 1.6 years) was calculated according to the different follow-up lengths for the different outcomes (*e.g.,* OGTT-derived measures and incident type 2 diabetes) according to the point at which the data were obtained.

### Clinical and laboratory measurements

The cross-sectional and follow-up studies of the METSIM cohort used identical protocols and similar clinical and laboratory measurements. All clinical and laboratory measurements were performed twice, at the baseline examination and at the follow-up visit. Height was measured to the nearest 0.5 cm and weight using a calibrated digital scale (Seca 877; Seca, Hamburg, Germany) to the nearest 0.1 kg. The BMI was calculated as the weight in kilograms divided by the height in square centimeters. The waist circumference was measured to the nearest 0.5 cm, with the average of two measurements taken after inspiration and expiration at the midpoint between the lowest rib and the iliac crest. The body composition was determined using bioelectrical impedance (Bioimpedance Analyzer Model BIA 101; Akern SrL, Florence, Italy) with the subjects in the supine position after a 12-hour overnight fast. The systolic and diastolic blood pressures (BPs) were calculated as the average of three measurements performed with the subject in the sitting position after a 10-minute rest using a mercury sphygmomanometer. Physical activity refers to leisure time physical activity (physically active, regular exercise for ≥30 minutes one or more times weekly vs physically inactive, occasional exercise, or no exercise). Smoking status was defined as current smoking (yes vs no).

The plasma glucose was measured using an enzymatic hexokinase photometric assay (Konelab Systems Reagents; Thermo Fisher Scientific, Vantaa, Finland). Insulin was determined by immunoassay (ADVIA Centaur Insulin IRI no. 02230141; Siemens Medical Solutions Diagnostics, Tarrytown, NY). HbA1c was analyzed using high-performance liquid chromatography on a Tosoh G7 glycohemoglobin analyzer (Tosoh Bioscience, Inc., San Francisco, CA), which was calibrated according to the Diabetes Control and Complications Trial standard. Total triacylglycerols (TAG), plasma free fatty acids, high-density lipoprotein (HDL) cholesterol, and low-density lipoprotein (LDL) cholesterol were measured using enzymatic colorimetric tests (Konelab Systems Reagents; Thermo Fisher Scientific, Vantaa, Finland). Serum concentrations of hs-CRP were assayed using kinetic immunoturbidimetry (near infrared particle immunoassay; IMMAGE Immunochemistry System; Beckman Coulter, Fullerton, CA) and plasma IL1-RA using a photometric immunoassay [enzyme-linked immunosorbent assay (ELISA)] method (Quantikine DRA00 Human IL-1RA; R&D Systems, Inc., Minneapolis, MN). Plasma adiponectin was measured using ELISA (human adiponectin ELISA kit; Linco Research, St. Charles, MI), and alanine aminotransferase by enzymatic photometric test (Konelab Systems Reagents; Thermo Fisher Scientific). Apolipoproteins A1 and B (ApoA1 and ApoB) were quantified using immunoturbidimetry (Konelab Systems Reagents; Thermo Fisher Scientific). Concentrations of glycoprotein acetyls (including mainly alpha-1-acid glycoprotein) were quantified from serum samples using a high-throughput proton nuclear magnetic resonance metabolomics platform, as previously described (29). Information on the GlycA level was available for 8716 of the 8749 nondiabetic men at the baseline study.

### Calculations

The trapezoidal method was used to calculate the glucose area under the curve (glucose AUC) using the OGTT samples collected at 0, 30, and 120 minutes. The Matsuda insulin sensitivity index (Matsuda ISI) (30) and early-phase insulin secretion index (InsAUC_0–30_/GluAUC_0–30_) were calculated as previously described (25). The disposition index (a marker of insulin secretion) was calculated as the Matsuda ISI × InsAUC_0–30_/GluAUC_0–30_.

### Statistical analysis

Statistical analyses were conducted using SPSS, version 19 (IBM Corp., Armonk, NY). All variables (except for age and LDL cholesterol) were logarithmically transformed for statistical analyses owing to their skewed distributions. The Spearman correlation was applied to explore the relationships between GlycA, IL-1RA, and hs-CRP and the clinical and metabolic traits in nondiabetic participants from the METSIM baseline study. The Fisher *r*-to-*z* transformation was used to compare the correlation coefficients. Linear regression analysis was used to evaluate GlycA, IL-1RA, and hs-CRP as predictors of changes in metabolic and clinical traits in a follow-up study. The results are presented as unstandardized coefficients in original units (B ± standard error) and standardized coefficient (*β*). Linear regression models were initially adjusted for the baseline level of the corresponding dependent trait, and an additional adjustment was done for age, follow-up time (in months), and BMI (except for the BMI, waist circumference, and fat mass). Models that included the systolic and diastolic BP were also adjusted for the use of antihypertensive medication at baseline, and the model that included LDL cholesterol was adjusted for statin medication use at baseline. Cox regression analysis was used to evaluate the ability of GlycA, IL-1RA, and hs-CRP to predict for incident type 2 diabetes, drug-treated hypertension, CVD events, and total mortality. Hazard ratios were calculated using standardized predictors, and hazard ratios with their 95% confidence intervals are presented. The Cox regression models were adjusted for age, BMI, smoking, physical activity, and LDL cholesterol levels at baseline. Principal component analysis (PCA) based on the correlation matrix with varimax rotation was used to reduce a set of three intercorrelated acute-phase proteins (GlycA, IL-1RA, and hs-CRP) to a smaller set of underlying principal components (PCs). The analysis yielded a single PC with an eigenvalue ≥1, explaining 52% of the total variance, with variable loadings of 0.760, 0.733, and 0.673 for hs-CRP, GlycA, and IL-1RA, respectively. The PC score obtained from the PCA was used in the Cox regression analysis as a predictor of disease outcomes. Bonferroni correction for multiple testing was performed to determine the statistical significance of the results (threshold for *P* value given for each analysis); *P* < 0.05 was considered to indicate nominal statistical significance.

## Results

### Baseline characteristics of study participants

Our present report included the data from 8749 Finnish men (mean age, 57.2 ± 7.1 years; BMI, 26.8 ± 3.8 kg/m^2^) without type 2 diabetes. The baseline GlycA level was measured in 8716 men (99.6%), and the IL-1RA and hs-CRP levels were measured in 8748 men (99.9%). The baseline cardiometabolic characteristics of the study participants relevant to the present study are summarized in Table 1.

### Correlations of acute-phase proteins with metabolic and clinical traits in the METSIM baseline study

GlycA correlated significantly (Bonferroni-adjusted *P* < 0.0009 for 19 traits × 3 acute-phase proteins) and positively with the baseline levels of IL-1RA (*ρ* = 0.246) and hs-CRP (*ρ* = 0.347). The correlation between the IL1-RA and hs-CRP levels was *ρ* = 0.276. All three acute-phase proteins correlated significantly and positively with measures of obesity (BMI, waist circumference, and fat mass), TAG, ApoB, glucose levels (FPG, 2hPG, and glucose AUC), alanine aminotransferase, and InsAUC_0–30_/GluAUC_0–30_, and negatively with the Matsuda ISI, disposition index, HDL cholesterol, and adiponectin level (Table 2). However, GlycA correlated more strongly than did the other two acute-phase proteins with TAG (*ρ* = 0.601), ApoB (*ρ* = 0.416), Matsuda ISI (*ρ* = −0.409), InsAUC_0–30_/GluAUC_0–30_ (*ρ* = 0.302), systolic and diastolic BP, and LDL and HDL cholesterol. IL-1RA correlated more strongly with BMI (*ρ* = 0.372), alanine aminotransferase (*ρ* = 0.249), and FPG (*ρ* = 0.168) than did the other two acute-phase proteins and hs-CRP showed the weakest correlation.

**Table 2.** **Spearman Correlations of Acute-Phase Proteins With Baseline Clinical and Metabolic Traits in Men Without Type 2 Diabetes in the METSIM Baseline Study**

Baseline Study Variable	GlycA, mmol/L			IL-1RA, µg/L			hs-CRP, mg/L		
	Men, n	*ρ*	*P* Value	Men, n	*ρ*	*P* Value	Men, n	*ρ*	*P* Value
Age, y	8716	0.022	0.040*^a^*	8748	−0.027	0.013*^a^*	8748	0.056	<0.0001*^b^*
BMI, kg/m^2^	8713	0.308	<0.0001*^b^*	8745	0.372*^c^*	<0.0001*^b^*	8745	0.332	<0.0001*^b^*
Waist, cm	8712	0.334	<0.0001*^b^*	8744	0.387	<0.0001*^b^*	8744	0.365	<0.0001*^b^*
Fat mass, %	8694	0.243	<0.0001*^b^*	8726	0.279	<0.0001*^b^*	8726	0.300	<0.0001*^b^*
Systolic BP, mm Hg	8716	0.177*^c^*	<0.0001*^b^*	8748	0.049	<0.0001*^b^*	8748	0.114	<0.0001*^b^*
Diastolic BP, mm Hg	8716	0.197*^c^*	<0.0001*^b^*	8748	0.117	<0.0001*^b^*	8748	0.136	<0.0001*^b^*
LDL cholesterol, mmol/L	8716	0.169*^c^*	<0.0001*^b^*	8745	−0.019	0.078	8745	0.108	<0.0001*^b^*
HDL cholesterol, mmol/L	8716	−0.344*^c^*	<0.0001*^b^*	8746	−0.268	<0.0001*^b^*	8746	−0.178	<0.0001*^b^*
TAG, mmol/L	8716	0.601*^c^*	<0.0001*^b^*	8748	0.240	<0.0001*^b^*	8748	0.171	<0.0001*^b^*
ApoA1, g/L	8716	−0.063	<0.0001*^b^*	8746	−0.133*^c^*	<0.0001*^b^*	8746	−0.095	<0.0001*^b^*
ApoB, g/L	8716	0.416*^c^*	<0.0001*^b^*	8746	0.120	<0.0001*^b^*	8746	0.185	<0.0001*^b^*
Adiponectin, μg/mL	8714	−0.169	<0.0001*^b^*	8746	−0.145	<0.0001*^b^*	8746	−0.101	<0.0001*^b^*
ALT, U/L	8716	0.163	<0.0001*^b^*	8748	0.249*^c^*	<0.0001*^b^*	8748	0.123	<0.0001*^b^*
FPG, mmol/L	8716	0.132	<0.0001*^b^*	8748	0.168*^c^*	<0.0001*^b^*	8748	0.060	<0.0001*^b^*
2hPG, mmol/L	8716	0.212	<0.0001*^b^*	8748	0.210	<0.0001*^b^*	8748	0.138	<0.0001*^b^*
Glucose AUC, mmol/L × min	8686	0.230	<0.0001*^b^*	8718	0.229	<0.0001*^b^*	8718	0.137	<0.0001*^b^*
InsAUC_0–30_/GluAUC_0–30_	8670	0.302*^c^*	<0.0001*^b^*	8702	0.243*^c^*	<0.0001*^b^*	8702	0.156*^c^*	<0.0001*^b^*
Matsuda ISI	8664	−0.409*^c^*	<0.0001*^b^*	8696	−0.353	<0.0001*^b^*	8696	−0.224	<0.0001*^b^*
Disposition index*^d^*	8664	−0.177	<0.0001*^b^*	8696	−0.185	<0.0001*^b^*	8696	−0.119	<0.0001*^b^*

Abbreviations: ALT, alanine aminotransferase; *ρ*, Spearman correlation coefficient.

^a^ *P* < 0.05 was considered to indicate nominal statistical significance.

^b^ Bonferroni-adjusted *P* < 0.00088 was considered to indicate statistical significance (0.05/19 traits × 3 acute-phase proteins).

^c^ Correlation coefficient was significantly (*P* < 0.05) different from correlation coefficients of other two acute-phase proteins with the same traits using Fisher *r*-to-*z* transformation.

^d^ Disposition index was calculated as Matsuda ISI × InsAUC_0–30_/GluAUC_0–30_.

### Association of acute-phase proteins with changes in metabolic and clinical traits in the METSIM follow-up study

We prospectively evaluated the associations of the three acute-phase proteins (GlycA, IL-1RA, and hs-CRP) with changes in selected cardiometabolic traits during the follow-up period in men without type 2 diabetes at baseline or diagnosed during the follow-up period (Table 3). The GlycA level at baseline was significantly (Bonferroni-adjusted *P* < 0.00093, 0.05/18 traits × 3 predictors) associated with increases in FPG, 2hPG, and glucose AUC and decreases in BMI and insulin secretion (disposition index) during the follow-up period in both models without and with covariates. IL-1RA was significantly associated with increases in waist circumference, fat mass, diastolic BP, InsAUC_0–30_/GluAUC_0–30_, and TAG and a decrease in the Matsuda ISI in both models. hs-CRP was significantly associated with increases in the fat mass, TAG, InsAUC_0–30_/GluAUC_0–30_, 2hPG, and glucose AUC and a decrease in Matsuda ISI in both models.

**Table 3.** **Association of Acute-Phase Proteins Measured at Baseline With Changes in Metabolic and Clinical Traits During 6.8-Year Follow-Up Study of Men Without Previously Diagnosed Type 2 Diabetes**

Trait at Follow-Up Study	GlycA, mmol/L						IL-1RA, µg/L						hs-CRP, mg/L					
	Men, n	B*^a^*	SE*^a^*	*β**^b^*	*P* Value*^c^*	*P* Value*^d^*	Men, n	*β**^a^*	SE*^a^*	*β**^b^*	*P* Value*^c^*	*P* Value*^d^*	Men, n	B*^a^*	SE*^a^*	*β**^b^*	*P* Value*^c^*	*P* Value*^d^*
BMI, kg/m^2^	5383	−0.336	0.098	−0.019	0.0006*^e^*	0.0004*^e^*	5400	0.213	0.143	0.015	0.008*^f^*	0.017*^f^*	5400	0.006	0.005	−0.004	0.480	0.989
Waist, cm	5383	−0.172	0.338	−0.003	0.640	0.458	5400	1.768	0.487	0.035	<0.0001*^e^*	<0.0001*^e^*	5400	0.039	0.017	0.021	0.003*^f^*	0.0003*^e^*
Fat mass, %	5336	0.833	0.327	0.032	0.002*^f^*	<0.0001*^e^*	5352	1.848	0.468	0.035	0.0006*^e^*	<0.0001*^e^*	5352	0.055	0.016	0.075	<0.0001*^e^*	<0.0001*^e^*
Systolic BP, mm Hg	5381	2.236	0.917	0.038	0.003*^f^*	0.845	5398	3.343	1.298	0.026	0.036*^f^*	0.001*^f^*	5398	0.104	0.046	0.058	<0.0001*^e^*	0.011*^f^*
Diastolic BP, mm Hg	5381	1.635	0.527	0.041	0.001*^f^*	0.055	5398	5.138	0.745	0.098	<0.0001*^e^*	0.0002*^e^*	5398	0.029	0.027	0.050	0.0001*^e^*	0.451
LDL cholesterol, mmol/L	5383	−0.156	0.047	−0.041	0.0005*^e^*	0.058	5399	−0.004	0.066	0.010	0.385	0.046*^f^*	5399	0.000	0.002	−0.024	0.039*^f^*	0.740
HDL cholesterol, mmol/L	5383	0.008	0.016	−0.006	0.460	0.869	5399	0.014	0.022	−0.013	0.109	0.727	5399	0.001	0.001	−0.011	0.169	0.994
TAG, mmol/L	5383	0.205	0.046	0.028	0.031*^f^*	0.073	5400	0.241	0.054	0.091	<0.0001*^e^*	<0.0001*^e^*	5400	0.005	0.002	0.071	<0.0001*^e^*	<0.0001*^e^*
ApoA1, g/L	5089	−0.036	0.012	−0.032	0.003*^f^*	0.060	5105	−0.047	0.017	−0.043	0.0001*^e^*	0.006*^f^*	5105	0.002	0.001	0.013	0.239	0.006*^f^*
ApoB, g/L	5089	0.024	0.014	0.007	0.552	0.229	5105	0.042	0.020	0.033	0.005*^f^*	0.012*^f^*	5105	0.002	0.001	0.028	0.014*^f^*	0.003*^f^*
Adiponectin, μg/mL	1299	−0.663	0.531	−0.046	0.025*^f^*	0.119	1303	−2.033	0.736	−0.073	0.0003*^e^*	0.014*^f^*	1302	0.026	0.037	−0.011	0.587	0.905
ALT, U/L	5383	1.630	0.892	0.020	0.053	0.313	5400	3.530	1.294	0.035	0.001*^f^*	0.046*^f^*	5400	0.027	0.045	0.008	0.469	0.989
FPG, mmol/L	5383	0.247	0.031	0.092	<0.0001*^e^*	<0.0001*^e^*	5400	0.102	0.045	0.023	0.049*^f^*	0.026*^f^*	5400	−0.001	0.002	0.052	<0.0001*^e^*	0.434
2hPG, mmol/L	5383	0.863	0.110	0.089	<0.0001*^e^*	<0.0001*^e^*	5400	0.850	0.156	0.072	<0.0001*^e^*	0.002*^f^*	5400	0.012	0.006	0.079	<0.0001*^e^*	<0.0001*^e^*
Glucose AUC, mmol/L × min	5341	70.392	8.058	0.100	<0.0001*^e^*	<0.0001*^e^*	5358	51.27	11.53	0.049	<0.0001*^e^*	0.294	5358	0.606	0.408	0.072	<0.0001*^e^*	0.0003*^e^*
InsAUC_0–30_/GluAUC_0–30_	5310	−0.667	1.117	−0.006	0.668	0.023*^f^*	5327	3.870	1.555	0.025	<0.0001*^e^*	0.003*^f^*	5327	0.112	0.055	0.020	<0.0001*^e^*	0.022*^f^*
Matsuda ISI	5303	−0.634	0.218	−0.034	0.0005*^e^*	0.043*^f^*	5320	−0.637	0.303	−0.061	<0.0001*^e^*	0.0003*^e^*	5320	−0.011	0.010	−0.066	<0.0001*^e^*	<0.0001*^e^*
Disposition index*^g^*	5303	−23.44	3.719	−0.084	<0.0001*^e^*	<0.0001*^e^*	5320	−17.54	5.352	−0.050	<0.0001*^e^*	0.145	5320	−0.205	0.189	−0.060	<0.0001*^e^*	0.004*^f^*

Abbreviations: ALT, alanine aminotransferase; *β*, standardized regression coefficient; SE, standard error.

^a^ Effect sizes (*β*, SE) are in original units.

^b^ *β* coefficients are standardized; *P* values and *β* coefficients were calculated using logarithmically transformed variables (except for age and LDL cholesterol).

^c^ *P* values adjusted for the baseline level of the corresponding dependent trait.

^d^ *P* values additionally adjusted for age, follow-up time, and BMI (except for BMI, waist, and fat mass); systolic and diastolic BP were additionally adjusted for antihypertensive medication at baseline, and LDL cholesterol for statin medication at baseline.

^e^ Bonferroni-adjusted *P* < 0.00093 was considered to indicate statistical significance (0.05/18 traits × 3 predictors).

^f^ *P* < 0.05 was considered to indicate nominal statistical significance.

^g^ Disposition index was calculated as Matsuda ISI × InsAUC_0–30_/GluAUC_0–30_.

### Association of acute-phase proteins with incident type 2 diabetes, new-onset drug-treated hypertension, CVD events, and total mortality

GlycA significantly (*P* < 0.003, 0.05/4 outcomes × 4 predictors) predicted for incident type 2 diabetes and CVD events in both unadjusted and adjusted (for age, BMI, smoking status, physical activity, and LDL cholesterol) Cox regression models (Table 4). IL-1RA predicted for total mortality and hs-CRP predicted for both total mortality and CVD events in the unadjusted model; however, these associations were attenuated to nominally statistically significant (*P* < 0.05) after adjustment for covariates. In addition, a single PC obtained from the PCA of all three acute-phase proteins was the strongest predictor of incident type 2 diabetes, CVD events, and total mortality in both unadjusted and adjusted models.

**Table 4.** **Association of Acute-Phase Proteins Measured at Baseline With Incident Type 2 Diabetes, New-Onset Drug-Treated Hypertension, CVD Events, and Total Mortality in a 6.8-Year Follow-Up METSIM Study**

Variable	GlycA				IL-1RA				hs-CRP				PC Including All Three Markers			
	N_TOTAL/EVENT_	HR (95% CI)	*P* Value*^a^*	*P* Value*^b^*	N_TOTAL/EVENT_	HR (95% CI)	*P* Value*^a^*	*P* Value*^b^*	N_TOTAL/EVENT_	HR (95% CI)	*P* Value*^a^*	*P* Value*^b^*	N_TOTAL/EVENT_	HR (95% CI)	*P* Value*^a^*	*P* Value*^b^*
Type 2 diabetes	8716/690	1.37 (1.29–1.46)	<0.0001*^c^*	<0.0001*^c^*	8748/692	1.18 (1.15–1.22)	<0.0001*^c^*	0.063	8748/692	1.07 (1.04–1.09)	<0.0001*^c^*	0.070	8714/688	1.66 (1.54–1.77)	<0.0001*^c^*	<0.0001*^c^*
Hypertension drug treatment	7147/224	1.17 (1.04–1.32)	0.009*^d^*	0.974	7170/225	1.07 (0.97–1.19)	0.183	0.131	7170/225	1.04 (0.97–1.12)	0.231	0.736	7145/224	1.31 (1.16–1.49)	<0.0001*^c^*	0.805
CVD events	8320/437	1.21 (1.12–1.32)	<0.0001*^c^*	0.003*^c^*	8351/439	1.09 (1.02–1.17)	0.015*^d^*	0.157	8351/438	1.06 (1.02–1.10)	0.001*^c^*	0.039*^d^*	8318/436	1.31 (1.20–1.44)	<0.0001*^c^*	0.0001*^c^*
Total mortality	8716/390	1.16 (1.06–1.27)	0.001*^c^*	0.077	8748/392	1.13 (1.07–1.20)	<0.0001*^c^*	0.007*^d^*	8748/392	1.08 (1.04–1.11)	<0.0001*^c^*	0.013*^d^*	8714/390	1.35 (1.22–1.48)	<0.0001*^c^*	0.0002*^c^*

Abbreviations: CI, confidence interval; HR, hazard ratio; N_TOTAL/EVENT_, number of participants and events.

Cox regression, HRs were calculated using standardized predictors (unit = standard deviation). Participants with type 1 diabetes (n = 25), type 2 diabetes (n = 763), newly diagnosed type 2 diabetes at baseline (n = 649), myocardial infarction, or stroke, or receiving antihypertensive treatment before the baseline study were excluded from the analyses.

^a^ Unadjusted *P* values.

^b^ *P* values adjusted for age, BMI, smoking, physical activity, and LDL cholesterol levels at baseline.

^c^ *P* < 0.0042 was considered to indicate statistical significance (given four outcomes and three inflammatory markers tested).

^d^ *P* < 0.05 was considered to indicate nominal statistical significance.

## Discussion

We evaluated the associations of three acute-phase proteins, GlycA, IL-1RA, and hs-CRP, at baseline with insulin secretion, insulin sensitivity, and insulin resistance-related traits, and with disease outcomes, including incident type 2 diabetes, drug-treated hypertension, CVD events, and total mortality in initially nondiabetic men from the 6.8-year follow-up portion of the METSIM cohort. These three acute-phase proteins differed in their associations with glucose metabolism-related parameters and the risk of type 2 diabetes, CVD, and total mortality. The important findings from our study are as follows: (1) the GlycA levels were associated with a decrease in insulin secretion and increases in glucose levels (FPG, 2hPG, and glucose AUC) compared with IL-1RA and hs-CRP levels, which were associated with a decrease in insulin sensitivity, and increases in waist circumference and/or fat mass, and TAG; (2) GlycA was the best predictor of incident type 2 diabetes and CVD events among the three acute-phase proteins; and (3) combining the three markers improved the prediction of type 2 diabetes, CVD events, and total mortality. These results suggest that all three acute-phase proteins capture early changes in cardiometabolic traits before the diagnosis of diabetes. Impaired insulin secretion and insulin resistance in multiple tissues are the two key mechanisms in the pathophysiology of type 2 diabetes. Several population-based studies have confirmed that low-grade systemic inflammation and increased levels of markers of inflammation such as hs-CRP, IL-6, tumor necrosis factor-*α*, and IL-1RA are already present in those with prediabetes (11, 31). Prospective studies have also shown that chronic low-grade inflammation precedes frank diabetes (7, 32). However, no large prospective studies have simultaneously evaluated several acute-phase proteins (*e.g.,* GlycA, IL-1RA, and hs-CRP) as predictors for changes in insulin secretion, insulin resistance, and insulin resistance-related traits. In a recent cross-sectional analysis that included 1225 participants from the Insulin Resistance Atherosclerosis Study (IRAS), GlycA, sialic acid (GlycB), and C-reactive protein levels were associated with insulin sensitivity but not with insulin secretion, measured by a frequently sampled intravenous glucose tolerance test and adjusted for insulin sensitivity (33). Our cross-sectional METSIM study included a sevenfold larger sample size and showed that GlycA, IL-1RA, and hs-CRP correlated significantly, not only with the Matsuda ISI, but also with the disposition index (insulin secretion adjusted for insulin sensitivity). Additionally, our prospective analysis demonstrated that the baseline levels of GlycA predicted for a decrease in the disposition index, but not in the Matsuda ISI, after adjustment for confounding factors. Our findings emphasize the need for large prospective population-based studies to investigate the role of inflammatory markers in metabolic changes over time.

Increased levels of circulating markers of inflammation, including acute-phase proteins, have been previously associated with the risk of type 2 diabetes and CVD in large cross-sectional (11, 34) and follow-up (5, 6, 9, 35) studies. GlycA, a glycan biomarker of low-grade inflammation, has been found to predict for type 2 diabetes in a 7.3-year follow-up analysis of the Prevention of Renal and Vascular End-stage Disease (PREVEND) study (12) and in a large population of initially healthy women (18). Moreover, GlycA levels have been associated with incident CVD events and CVD mortality (19, 21). However, none of the previous follow-up studies has simultaneously evaluated the associations of multiple acute-phase proteins with type 2 diabetes, several adverse cardiovascular outcomes, and total mortality.

We have demonstrated that GlycA is the strongest predictor of an increase in glycemia (FPG, 2hPG, and glucose AUC), incident type 2 diabetes, and CVD events after the adjustment for confounding factors among the three acute-phase proteins. Additionally, GlycA correlated strongly with several cardiovascular and diabetes risk factors at baseline (including TAG, Matsuda ISI, and LDL and HDL cholesterol), and significantly predicted for a decrease in insulin secretion during the follow-up period in men without previously diagnosed diabetes. The measurement of GlycA using proton nuclear magnetic resonance incorporates the signal from several acute-phase reactants and immunologic proteins and, therefore, represents an integrated plasma biomarker. Furthermore, GlycA reflects an altered enzymatic glycosylation pattern, which plays an important role in the pathogenesis of low-grade inflammation–related diseases such as atherosclerosis, metabolic syndrome, and diabetes (36–39). Altogether, GlycA could reflect the risk associated with a summation of multiple pathways of low-grade systemic inflammation and, thus, predict for early changes in glucose metabolism and cardiometabolic traits and, consequently, in the risk of type 2 diabetes and CVD, as demonstrated in our study. However, additional studies are needed to explore in detail the pathways underlying the associations we observed in our study.

IL-1RA and hs-CRP were not associated with an increased risk of incident type 2 diabetes after adjustment for confounding factors in our 6.8-year follow-up study but nominally significantly predicted total mortality. Also, hs-CRP nominally significantly predicted CVD events. Accordingly, IL-1RA and hs-CRP were predominantly associated with adverse changes in adiposity (waist circumference and fat mass) and insulin sensitivity but not in insulin secretion (disposition index) during the follow-up period. Elevated levels of IL-1RA and hs-CRP are generally considered to indicate a proinflammatory state. The major source of circulating IL-1RA is adipose tissue (10, 40), and increased levels of IL-1RA are often found in obese individuals. IL-1 family members are known to play a role in the pathology of type 2 diabetes by triggering obesity-induced inflammation (10, 40). hs-CRP has also been related to adiposity and insulin resistance (3, 33), which might explain the association of IL-1RA and hs-CRP with adverse changes in adiposity and insulin sensitivity in our prospective study.

The PCA that included all three acute-phase proteins resulted in a single PC that was a better predictor of incident type 2 diabetes, CVD events, and total mortality than any of the markers used individually. This also suggests that the three markers capture different aspects of low-grade inflammation and that combining them improves the risk prediction.

The strengths of our study were that it used data from a large well-characterized cohort of men with a long follow-up period. Furthermore, insulin sensitivity and secretion were measured by indexes validated against reference standard measurements. One limitation of our study was that, because we included only middle-aged and elderly Finnish men, we could not ensure the applicability of our results to women or other populations. Therefore, attempts should be made to replicate our results in women, other ethnic groups, and other age groups.

## Conclusions

In the present study, we have demonstrated that the three acute-phase proteins examined differentially predicted for changes in metabolic parameters and the risk of type 2 diabetes and cardiovascular outcomes. GlycA predicted for impaired insulin secretion, incident type 2 diabetes, and CVD events and adverse changes in glycemia during the follow-up period. IL-1RA and hs-CRP were associated with an increase in insulin resistance and obesity markers during the follow-up period, suggesting that these three markers capture different aspects of low-grade inflammation. The combined effect of all three acute-phase proteins improved the prediction of incident type 2 diabetes, CVD events, and total mortality compared with the markers used individually.
